# Motor control characteristics of upper limbs in response to assistive forces during bilateral tasks

**DOI:** 10.1371/journal.pone.0245049

**Published:** 2021-01-07

**Authors:** Yuanyuan Wang, Ping Yeap Loh, Satoshi Muraki

**Affiliations:** 1 Graduate School of Science and Engineering, Chiba University, Chiba, Japan; 2 Department of Human Science, Faculty of Design, Kyushu University, Fukuoka, Japan; University of Pittsburgh, UNITED STATES

## Abstract

Most research on power assist suits (PASs) that concerned PAS-human interactions has used human physical reactions as criteria to evaluate the mechanical function, however, with minimal emphasis on human reactions in response to PASs. In this study, we focused on the physiological responses of the upper limbs including muscle activity of the biceps brachii and the triceps brachii, co-activation, force steadiness (CV) and rated perceived exertion (RPE) to various patterns of bilateral assistive force, such as unilateral assistance (L0% & R67% [% = percentage of workload force, L = left arm, R = right arm], L67% & R0%, L0% & R33%, L33% & R0%), symmetrical (L0% & R0%, L33% & R33%, L67% & R67%) and asymmetrical bilateral assistance (L33% & R67%, L67% & R33%), during bilateral isometric force-matching tasks. The results showed a similar muscular response of the two arms to bilateral assistive conditions, and the muscle activity of the arm that was being observed decreased only when the assistive force that applied on itself increased, indicating that both arms may have adopted similar but independent motor control mechanisms to acclimate to the bilateral assistive forces. Comparison between the two unilateral assistances (L0% & R33% and L33% & R0%) and the two asymmetrical bilateral assistances (L33% & R67%, L67% & R33%) showed no significant differences in muscular responses, CV and RPE, indicating that bilateral assistances with bilateral interchanged assistive levels may be equally effective regardless of which arm the higher assistive force is applied to. Comparison between unilateral and symmetrical assistive conditions that have similar overall workloads (L67% & R0%, L33% & R33%, L0% & R67%) showed a lower CV and RPE score at symmetrical assistance compared with unilateral assistance, suggesting that assisting both arms with the same level simultaneously improves task performances compared with applying the assistive force to only one arm.

## Introduction

Power assist suits (PASs) are wearable machines that augment human physical capabilities and have been applied to many fields. For example, as a replacement for therapists, PASs can be used as rehabilitation devices that provide assistive technology to move impaired limbs during physical training [1, 2] or as assistive devices for daily use. In the industrial sector, PASs have been designed to perform heavy lifting and can augment the strength of limbs and help maintain correct back posture while bending down to perform lifts [3].

Recently, research on PAS has focused not only on mechanical development but also on human responses. Hayashi, Kawamoto, & Sankai (2005) evaluated the assistive method of the hybrid assistive limb robot suit by analyzing the myoelectric activities of extensor and flexor muscles [4]. Their experiments showed a significant reduction of muscle activities, thus confirming the effectiveness of the device and the assistive method. Several studies on personal lifting assistive devices measured the activity of the back muscles when using the developed device [5, 6]. Nevertheless, most studies used human physical reactions as the criteria to evaluate the mechanical function, with minimal research on human-PAS interactions. Humans are capable of modulating their physical outputs based on muscle tension feedback and their own experiences. However, when an external assistive force is applied, the physical outputs can be different from those expected, and this gap might disrupt motor control systems [7]. Accordingly, human physiological interactions with robotic assistive devices must be considered during PAS development.

This research focused on PASs for upper limbs since well-functioning upper limbs are crucial to a higher quality of life. Many daily activities are performed as a result of the coordination of both upper limbs (bilateral). Such bilateral movements often require some degree of coordination between the limbs (inter-limb) and limb segments (intra-limb) [8]. These coordination patterns arise from a strong natural tendency to synchronize the limbs, resulting in preferred modes of coordination [9]. With respect to inter-limb coordination, the preferred modes have been identified as in-phase and anti-phase. The in-phase mode involves a mirrored symmetrical limb movement associated with the same timing of activity onset in homologous muscle groups (i.e., flex or extend both arms simultaneously). The anti-phase mode requires asymmetrical limb movements in which non-homologous muscle groups are simultaneously activated (i.e., one arm flexes while the other extends) [10, 11]. Although both coordination modes synchronize the movement of both arms, the in-phase mode movements show higher accuracy and stability than the anti-phase modes, especially at a high movement frequency [12, 13].

The majority of previous studies examined two fundamental patterns of bilateral movements: Symmetrical patterns, where both arms share identical tasks, and asymmetrical patterns, where each arm is assigned an independent movement. Movements of the two arms usually differ in terms of timing, phase of movement period, muscle groups involved, and force levels. For instructed asymmetrical patterns, these studies have observed bilateral coupling, i.e., limb movements deviate from the original (instructed) patterns towards a synchronized pattern. Depending on the characteristics of the movement task, limb movements showed temporal coupling, phase coupling, homologous coupling, and force coupling, respectively [14, 15]. Furthermore, bilateral interference, which is a deterioration in performance caused by coupling, has been observed namely in asymmetrical tasks; for example, bilateral interference occurs due to asymmetry in task demand, where the arm that is assigned the easier task suffers greater interference than the contralateral arm that performs the more complicated task [16, 17].

Hand dominance may also affect bilateral coordination because daily preferential use may alter the motor unit properties of skeletal muscles. It has been reported that during submaximal isometric flexion of the first dorsal interosseous muscle, motor units in the dominant hand show lower average firing rates and recruitment thresholds compared with the non-dominant hand, which suggest higher mechanical effectiveness of motor units in the dominant hand [18]. In addition, previous studies suggested different motor control strategies [19] and physiological characteristics of the two arms; for example, the dominant limb might be superior to the non-dominant limb in terms of muscular strength and dexterity [20, 21].

Generally, three factors (inter-limb coordination pattern, bilateral task pattern, and handedness) can influence the performance of both arms during bilateral movements. On the other hand, PAS can provide support during bilateral movements in multiple ways; for instance, by providing assistive force to a non-dominant underperforming arm or providing different degrees of support to the dominant and non-dominant arms (unilateral and asymmetrical assistance). It is also possible to provide the same degree of assistive force to both arms (symmetrical assistance). Currently, it remains unclear how the two arms coordinate with each other or with the external assistive force during various patterns of bilateral assistance. Also, which assistive patterns result in better task performances remains to be verified. Resolutions of these research tasks might provide insights into PAS control methods for bilateral movements. Therefore, we aimed to investigate the muscle activity of the biceps brachii (BB) and the triceps brachii (TB) in both arms, force steadiness (coefficient of variation [CV]), and rated perceived exertion (RPE) during various patterns of assistive force, including unilateral assistance and symmetrical and asymmetrical bilateral assistances, when performing bilateral isometric elbow flexion. We aimed to determine.

1. whether or not bilateral differences (effect of handedness) could be observed in the assisted bilateral movements. Based on the superior motor unit properties of the skeletal muscles of the dominant arm, we hypothesized that the dominant arm would utilize the assistive force more efficiently and show a lower level of muscle activity than the non-dominant arm (hypothesis 1).
2. whether or not the change in the assistive level of the contralateral arm affected the performance of the arm that was being observed (the objective arm). We hypothesized that the muscle activity of the objective arm that is assisted at a constant level would vary with the change in the assistive level of the contralateral arm owing to the different degrees of task asymmetry (hypothesis 2).
3. the most effective assistive pattern. We hypothesized that symmetrical bilateral assistance would result in a lower CV and RPE than would unilateral and asymmetrical bilateral assistance, as symmetrical assistance might more easily achieve the in-phase coordination of the two arms, and assisting the two arms with the same level would minimize the bilateral interference (hypothesis 3).

## Methods

### Participants

In total, 13 healthy right-handed male university students (Table 1) participated in this study. Arm dominance was determined by the Edinburgh Handedness Inventory [22]. All participants gave written informed consent and the experiment was approved by the Ethics Committee of the Faculty of Design, Kyushu University, Japan.

**Table 1:** Characteristics of participants (n = 13).

		Minimum	Maximum	Mean ± SD
**Age (years)**		23.0	29.0	24.2 ± 1.8
**Height (cm)**		164.1	182.3	174.6± 6.5
**Weight (kg)**		53.4	81.8	65.9 ± 8.6
**Left arm (cm)**	**Forearm length**	21.0	25.2	23.0 ± 1.3
	**Upper arm length**	29.9	34.5	32.3 ± 1.3
**Right arm (cm)**	**Forearm length**	21.4	25.5	23.3 ± 1.5
	**Upper arm length**	29.9	34.3	32.1 ± 1.4

SD: Standard deviation.

### Experimental setup

**Fig 1:**
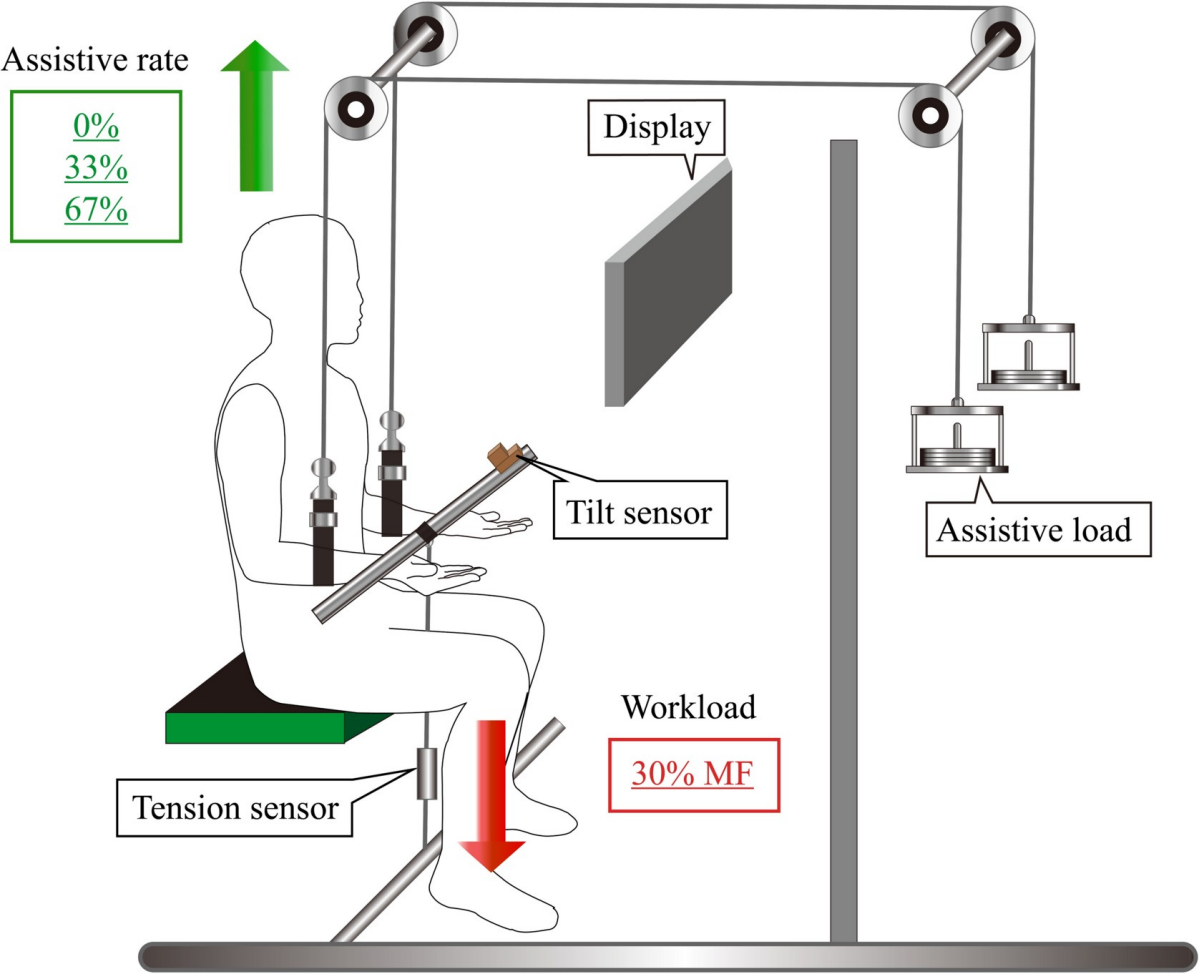
Experimental setup for bilateral isometric elbow flexion with assistance. MF: Maximum bilateral force.

We mimicked the scenario of PAS-assisted weight holding using the experimental setup shown in Fig 1. Participants sat upright in an armless chair, leaned on a backrest with both arms positioned at 90˚ elbow flexion with the forearm in supination and palm facing upward. A 21-inch monitor was placed approximately 0.7 meters in front of the participants.

A straight, rigid rod with a tension sensor fixed in the center was secured at the participants’ wrists to connect the two forearms to the tension sensor that measured force production. The maximum height of the rod was adjusted for each participant to ensure that both their elbows were maintained at 90˚ elbow flexion during force production. A tilt sensor was attached to the rod to record its tilt angle and to confirm whether the two arms were kept at the same level during the isometric bilateral tasks. Assistive force was applied to each forearm using an algorithm with assistive loads and a fixed-pulley system: one end of the strap threading through the two pullies was secured to the middle of each forearm, and the other end was connected to the assistive load. Therefore, the assistive force equaled the gravity force of the load and had a direction opposite to the workload.

**Fig 2:**
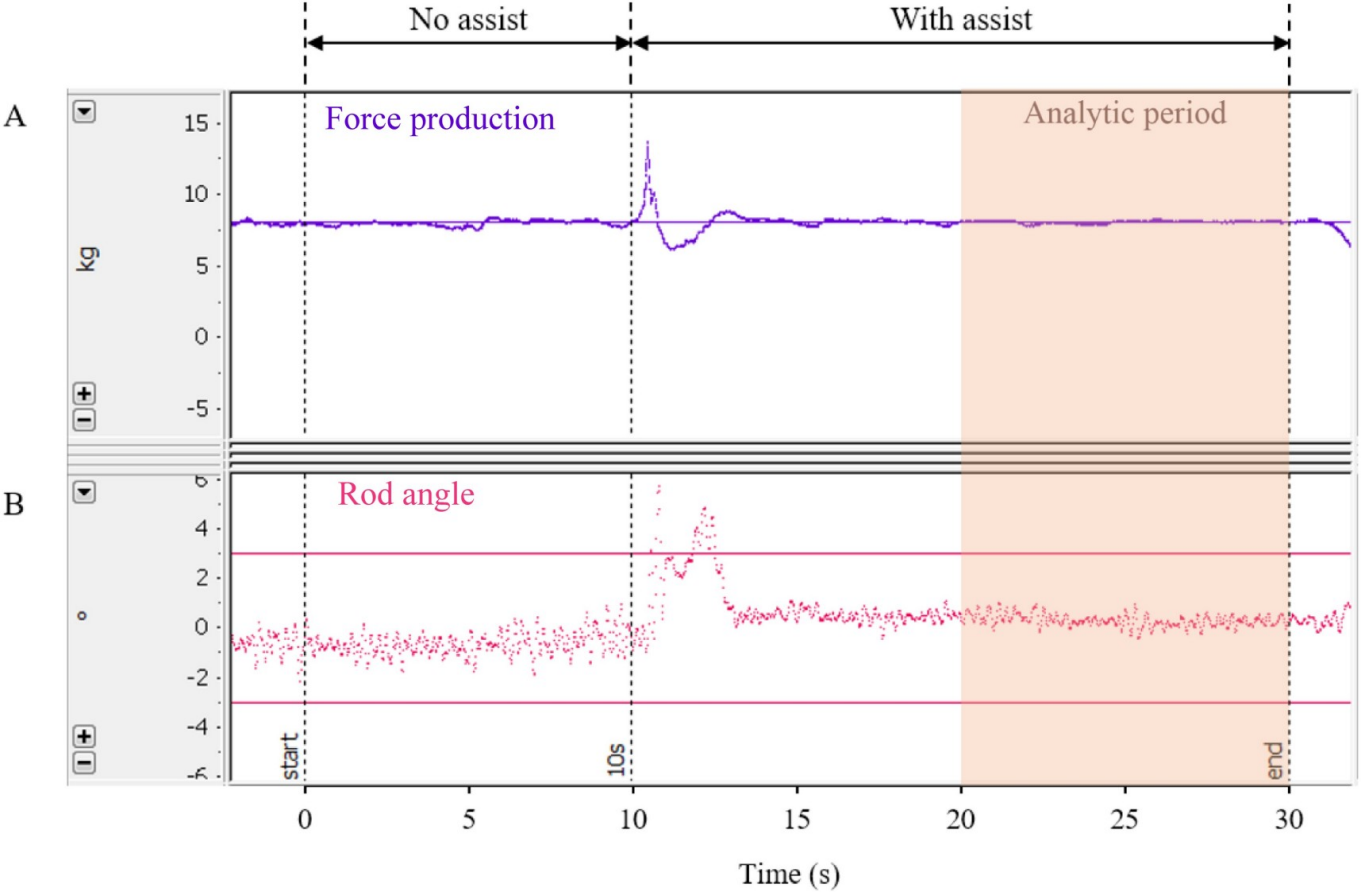
Visual feedback displayed on the monitor. (A) Force exertion, (B) Angle of the rod.

A monitor was used to display lines representing the target force and the force produced by both arms (Fig 2A). The tilt angle of the rod measured by the tilt sensor related to the target range was also displayed on the monitor in real-time to provide participants with visual feedback (Fig 2B). It was difficult to maintain the rod in a horizontal position since both arms inevitably shifted during force production [23, 24]. Thus, participants were instructed to adjust the angle between -3˚ and 3˚ during the experimental tasks.

### Experimental conditions

Workload was determined based on the maximum bilateral force (MF) produced by both arms simultaneously (30% MF) (Eq 1). Workload sustained by each arm during the bilateral task (*Workload*_*left*(*right*)_) was determined in terms of the maximum unilateral force production (MVF) of each arm (Eq 2). Assistive load for each arm was set at three levels that equaled 0%, 33%, and 67% of *Workload*_*left*(*right*)_ (Eq 3), which was calculated by the following equations:

Workload=30%×MF (1)

$$Workload_{left(right)}=\frac{MVF_{left(right)}}{MVF_{left}+MVF_{right}}\times Workload$$ (2)

$$Assistive\;load_{left(right)}=x_{left(right)}\%\times Workload_{left(right)}$$ (3)

$$x_{left},x_{right}=0,33,67$$

These calculations partially mimicked the muscle-exertion-based feedback control algorithm of a PAS. Combinations of the assistive levels of the left (L) and right (R) arms resulted in nine different bilateral assistive conditions with three assistance patterns: (1) Unilateral assistance: assistive force only applied to one arm (L0% & R33% [% = percentage of workload force], L0% & R67%, L33% & R0%, L67% & R0%); (2) symmetrical bilateral assistance: the same level of assistive force applied to both arms simultaneously (L33% & R33%, L67% & R67%), or no assistive force applied (L0% & R0%); and (3) asymmetrical bilateral assistance: different levels of assistive force applied to both arms (L33% & R67%, L67% & R33%). For example, if a participant had a maximum bilateral force of 35 kg (Workload = 10.5 kg (Eq 1)) and the unilateral force exertion ability of the left and the right arms were 17 and 19 kg (bilateral deficit was observed in this study), the workload of the left and right arms were 4.96 and 5.54 kg (Eq 2), respectively. Based on these workloads, the following assistive conditions were tested: (1) L0% & R0%, assist both arms with 0 kg; (2) L0% & R33%, assist the left arm with 0 kg and the right arm with 1.83 kg; (3) L0% & R67%, assist the left arm with 0 kg and the right arm with 3.71 kg; (4) L33% & R0%, assist the left arm with 1.64 kg and the right arm with 0 kg; (5) L33% & R33%, assist the left arm with 1.64 kg and the right arm with 1.83 kg; (6) L33% & R67%, assist the left arm with 1.64 kg and the right arm with 3.71 kg; (7) L67% & R0%, assist the left arm with 3.32 kg and the right arm with 0 kg; (8) L67% & R33%, assist the left arm with 3.32 kg and the right arm with 1.83 kg; and (9) L67% & R67%, assist the left arm with 3.32 kg and the right arm with 3.71 kg. The nine trials were randomly performed and rest periods, at least 180 s in length, were provided between consecutive trials.

### Experimental protocol

Participants were asked to avoid high-intensity exercises that could cause muscle fatigue before the day of the experiment. Before the experiment, participants changed clothes into short-sleeved shirts and short pants that we provided. The experimental session involved maximum isometric voluntary contraction (MVC) tasks, bilateral MF tasks, and bilateral force-matching tasks. MVC tasks that were performed on each arm included MVF measurements of the BB muscle and the maximum amplitude of surface electromyography (sEMG) of the BB and the TB of each arm. The bilateral MF task measured the MF generated by both arms simultaneously. The bilateral force-matching task (with assistance) was performed immediately after the bilateral MF task.

#### MVC task

Participants were instructed to maintain their arms positioned at 90˚ elbow flexion with their hands in supination. To measure the MVC of the BB, a strap connected to a ground-fixed tension sensor was placed on their wrists. Each participant was asked to pull against the tension sensor with maximum effort for 5 s. To measure the MVC of the TB, participants were asked to press down against the armrest, the height of which was adjusted to maintain the elbow joint angle at 90˚, with maximum effort for 5 s. Three MVC trials were performed for each muscle with 60 s of rest interspersed between each trial to avoid fatigue. Maximum tension and sEMG amplitude were quantified by averaging data over a 3 s interval where the peak force was generated. The mean sEMG amplitude of three trials was used to normalize sEMGs during force-matching tasks. The MVC tasks for both arms were performed in random sequences.

#### Bilateral MF task

During the bilateral MF task, participants were instructed to maintain their arms positioned at 90˚ elbow flexion. A rigid rod connected to a ground-fixed tension sensor was attached to both wrists. Each participant was asked to use both arms to pull against the rod with maximum effort for 5 s, while the tilt angle of the rod was maintained within -3˚ to 3˚. If the mean angle of 5 s exceeded the target range, the trial was considered a failure and was repeated. At least three MF trials were performed, and 60 s of rest was interspersed between each trial to avoid fatigue. The bilateral MF was quantified by averaging data over a 3 s interval, and the mean MF was the average of three trials.

#### Bilateral force-matching task with assistance

During the force-matching task, participants were instructed to maintain a target force (workload) that equaled 30% of the MF, which is adequate for observing muscle response without causing fatigue, and a rod tilt angle range of -3˚ to 3˚ for 30 s with both arms maintained at 90˚ elbow flexion. The bilateral force-matching task consisted of two phases: 1) Between 0–10 s, no assistive force was applied; and 2) between 11–30 s, assistive loads were applied to both arms simultaneously (Fig 2). Each participant performed nine trials of the bilateral force-matching task assisted with different bilateral assistive conditions in random sequence and rest periods, at least 60 s in length, were provided between consecutive trials.

### Measurements

#### sEMG recordings

sEMG signals of the BB and TB muscles were detected using bipolar Ag/AgCl pre-gelled electrodes (34 mm diameter, 25 mm inter-electrode distance, Ambu Inc., United States). The location of the BB and TB muscles were palpated, and the skin above the muscle was gently scrubbed with an abrasive gel and cleaned with alcohol to enhance signal conductivity. The electrode location and orientation were determined according to the SENIAM recommendations for sensor location [25]. Reference electrodes were attached over the head of the radius and the acromion. Raw sEMG signals were amplified (×1,000) using Bio-amp ML 132 (AD Instruments, Australia), recorded at a sampling frequency of 1,000 Hz, and filtered by a band-pass with a cutoff frequency ranging from 15 to 500 Hz. sEMG signals were digitized by an A/D converter (PowerLab 16/30, AD Instruments, Australia), exported to a personal computer and processed using LabChart v7.1.1 (AD Instruments, Australia) for further analysis. sEMG digital signals were full-wave rectified and sEMG amplitude for each second was calculated as the average of 1,000 samples.

#### Tension recordings

Tension during the tasks was recorded using a tension sensor (range 0–100 kg, T.K.K. 1269f, Takei Scientific Co., Japan), and the tension signals were amplified using a strain amp TSA-210 (accuracy: below ±0.2%FS, T.K.K. 1268b, Takei Scientific Co., Japan).

#### Tilt angle

The rod tilt angle was recorded using a linear tilt sensor (sensitivity: 25mV/° ± 3%, D5R-L02-60, OMRON Co., Japan). A negative degree value indicated that the right side of the rod was higher than the left side, and a positive value meant that the left side was higher.

Synchronously with EMG signals, the analog tensions and tilt angles were converted into digital signals using an A/D converter, sampled at a frequency of 1,000 Hz. The baseline noise of the tension sensor and the linear tilt sensor were filtered with a low-pass filter at 30 Hz in LabChart v7.1.1 (AD Instruments Pty. Ltd., Australia) prior to the analysis.

#### Perceived exertion

The RPE during force-matching tasks was evaluated using Borg’s CR-10 scale, which has a range from 0 to 10, with 0 being no muscle effort and 10 being maximum muscle effort [26]. Participants rated their exertion immediately after each experiment trial.

### Data analysis

#### Muscle activity of BB and TB (%MVC)

The sEMG amplitudes of the BB and TB muscles measured during the force-matching trials were divided by the maximum sEMG of the MVCs for normalization. The muscle activity was evaluated using normalized sEMGs (%MVC).

#### Force steadiness

Force steadiness was evaluated through fluctuations in isometric force production, which were quantified using the coefficient of variation (CV) of tension.

#### Co-activation

The level of co-activation during the bilateral force-matching tasks was calculated using Rudolph et al.’s method [27] with the following equation:

$$\text{Co-activation}=\frac{\text{\%}MVC_{TB}}{\text{\%}MVC_{BB}}\times \left(\text{\%}MVC_{BB}\text{+\%}MVC_{TB}\right)$$

The ratio of muscle activity found in the antagonist muscle (TB) and the agonist muscle (BB) was multiplied by the sum of the muscle activity of the two muscles. This method is able to estimate the relative activation of the pair of antagonistic muscles and weaken the influence of the agonist muscle when its muscle activity is greatly different from (often much larger than) the antagonist one, especially in this study, where the assistive force was aimed at reducing the muscle activity of the agonistic muscle (BB).

#### Analytic period

The analytic period was defined as the 10 s interval from the 21^st^ s when force production began to stabilize near the end of the force-matching task (emphasized by the shaded region in Fig 2). This interval is also referred to as a steady period.

### Statistical analysis

Task performance measurements, including %MVC_BB_, %MVC_TB_, co-activation, CV, and RPE, were analyzed using analysis of variance (ANOVA) tests to determine significant differences between arms and among bilateral assistive conditions, with a significance level set at p < 0.05. All statistical analyses were performed using SPSS version 25.0. (IBM Corp. Released 2017). All data are reported as mean ± standard deviation (SD).

#### Statistical analysis for bilateral difference

The nine assistive conditions can be divided into 6 pairs that have interchanged assistive levels of the two arms, namely 3 symmetrical assistive conditions, 2 pairs of unilateral conditions, and 1 pair of asymmetrical conditions (Table 2). A two-way repeated-measures ANOVA (2 arms × 9 bilateral assistive conditions) was used to test hypothesis 1: The dominant arm would show a lower level of muscle activity than the non-dominant arm. The violation of sphericity was examined by Mauchly's test and Greenhouse-Geisser correction was used for repeated-measures ANOVA. Post hoc pairwise Bonferroni-corrected comparison was used to compare the %MVC_BB_, %MVC_TB_, and co-activation between the arms that were assisted at the same level in these pairs (i.e., the difference between the left arm in L33% & R67% and the right arm in L67% & R33%), resulting in 9 bilateral comparison pairs in total (Table 2).

**Table 2:** Comparison pairs for investigating bilateral differences.

	Symmetrical			Unilateral								Asymmetrical			
Assistive Conditions	L0 & R0	L33 & R33	L67 & R67	L0 & R33		L33 & R0		L0 & R67		L67 & R0		L33 & R67		L67 & R33	
**Bilateral comparison pairs**	Left v. Right	Left v. Right	Left v. Right	Left	v.		Right	Left	v.		Right	Left	v.		Right
				Right	v.		Left	Right	v.		Left	Right	v.		Left

L: Left arm (non-dominant arm), R: Right arm (dominant arm), v.: Versus.

#### Statistical analysis for the interference of bilateral assistive levels

A two-way repeated-measures ANOVA (3 left assistive levels × 3 right assistive levels) was used to test hypothesis 2: The muscle activity of the objective arm that is assisted with a constant level should vary with the change in the assistive level of the contralateral arm. Post hoc pairwise Bonferroni-corrected comparison was used to compare the %MVC_BB_, %MVC_TB,_ and co-activation of the objective arms at assistive conditions that apply the same assistive level on the objective arm but different assistive levels on the contralateral arm, and vice versa. This analytical method was also used to investigate the effect of the bilateral assistive level changes on CV and RPE.

#### Statistical analysis for CV and RPE

The normality of RPE results were tested by Shapiro-Wilk test, which showed that RPE under assistive conditions of L0% & R0% (W [13] = 0.925, p > 0.05), L33% & R0% (W [13] = 0.928, p > 0.05), L33% & R33% (W [13] = 0.901, p > 0.05), L33% & R67% (W [13] = 0.935, p > 0.05) were normally distributed, whereas the other conditions were not. Post hoc tests would involve both normally and non-normally distributed assistive conditions; therefore, all RPE data were log-transformed after examining their skewness and kurtosis. The transformed RPE data were then back-transformed and reported as mean ± SD. Subsequently, we used a one-way repeated-measures ANOVA (9 bilateral assistive conditions) with post hoc pairwise Bonferroni-corrected comparison to compare the CV and RPE between assistive conditions.

To determine the preferable bilateral assistance (hypothesis 3), we compared two unilateral assistive conditions and one symmetrical bilateral assistive condition, namely L0% & R67%, L33% & R33%, and L67% & R0%, which have approximately the same overall workload. We analyzed the overall (summed) muscle activity of the left and right arms since the emphasis of this case analysis was on the overall performance of both arms. A one-way repeated-measures ANOVA (3 assistive conditions) was used to determine if there were differences among the 3 assistive conditions with respect to the overall %MVC_BB_ and %MVC_TB_.

## Results and discussion

This section consists of two parts: (1) Results and discussion of the overall results of nine bilateral assistive conditions with respect to hypotheses 1 and 2; (2) Results and discussion of case analysis with respect to hypothesis 3.

### Overall results and discussion of nine bilateral assistive conditions

#### Temporal changes in bilateral muscle activity

**Fig 3:**
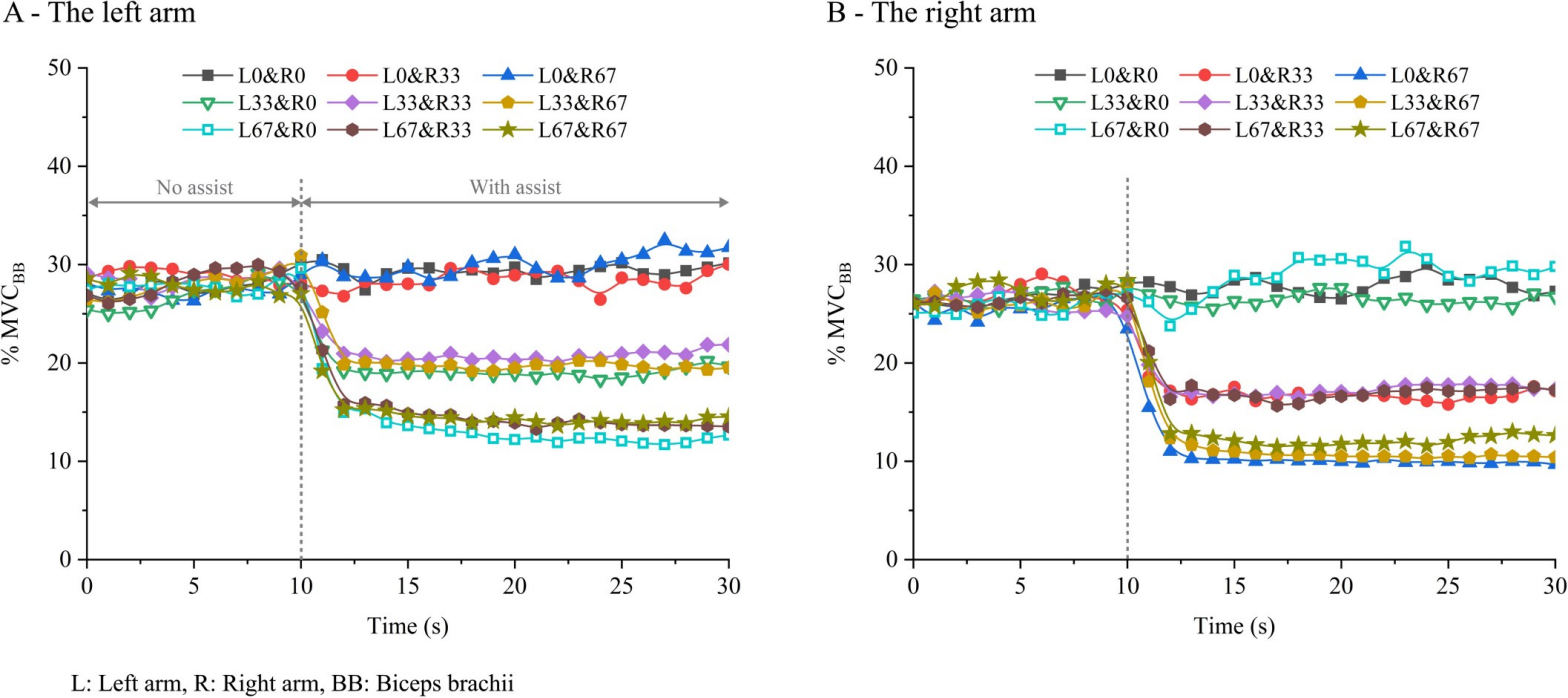
Temporal changes in bilateral biceps brachii muscle activity.

The %MVC_BB_ temporal changes of the two arms at different assistive levels are shown in Fig 3. The %MVC_BB_ of both arms showed similar tendencies that decreased with the intensity of the assistive force that was applied to itself. Bilateral %MVC_TB_ also showed similar temporal changes to that observed in %MVC_BB_, with much smaller values (Fig A in S1 Fig).

#### Steady period results

Statistical analysis for bilateral difference (two-way ANOVA: [2 arms × 9 bilateral assistive conditions]) revealed no significant main effect of the arm condition on %MVC_BB_ (F[1, 12] = 1.44, p = 0.254), %MVC_TB_ (F[1, 12] = 0.98, p = 0.342), and co-activation (F[1, 12] = 0.96, p = 0.347). Accordingly, no bilateral difference in muscular responses was found between the two arms. The results of the analysis of the interference of the bilateral assistive levels (two-way ANOVA: [3 left assistive levels × 3 right assistive levels]) are shown in Table 3.

**Table 3:** Statistical results of the bilateral interference.

		Left assist level	Right assist level	Left assist level × Right assist level
**%MVC**_**BB**_	**Left**	F [1.37, 16.43] = 118.33, p < 0.01, ε = 0.69	F [2, 24] = 2.28, p = 0.124	F [4, 48] = 2.28, p = 0.292
	**Right**	F [2, 24] = 1.88, p = 0.174	F [1.34, 16.11] = 78.41, p < 0.01, ε = 0.67	F [1.59, 19.09] = 1.43, p = 0.260, ε = 0.40
**%MVC**_**TB**_	**Left**	F [1.02, 12.24] = 13.46, p < 0.01, ε = 0.51	F [2, 24] = 1.73, p = 0.200	F [4, 48] = 0.79, p = 0.541
	**Right**	F [2, 24] = 1.90, p = 0.172	F [1.02, 12.26] = 23.70, p < 0.01, ε = 0.51	F [4, 48] = 1.44, p = 0.258
**Co-activation**	**Left**	F [1.01, 12.14] = 10.18, p < 0.01, ε = 0.51	F [1.33, 15.91] = 1.10, p = 0.331, ε = 0.66	F [4, 48] = 1.36, p = 0.264
	**Right**	F [2, 24] = 1.55, p = 0.233	F [1.01, 12.16] = 20.36, p < 0.01, ε = 0.51	F [2.11, 25.36] = 1.66, p = 0.209, ε = 0.53
**CV**		F [2, 24] = 8.21, p < 0.05	F [2, 24] = 19.45, p < 0.01	F [2.00, 23.98] = 6.28, p < 0.01, ε = 0.50
**RPE**		F [2, 24] = 36.61, p < 0.01	F [2, 24] = 47.73, p < 0.01	F [4, 48] = 5.96, p < 0.01

MVC: Maximum voluntary contraction, BB: Biceps brachii, TB: Triceps brachii, CV: Force steadiness, RPE: Rated perceived exertion.

**Fig 4:**
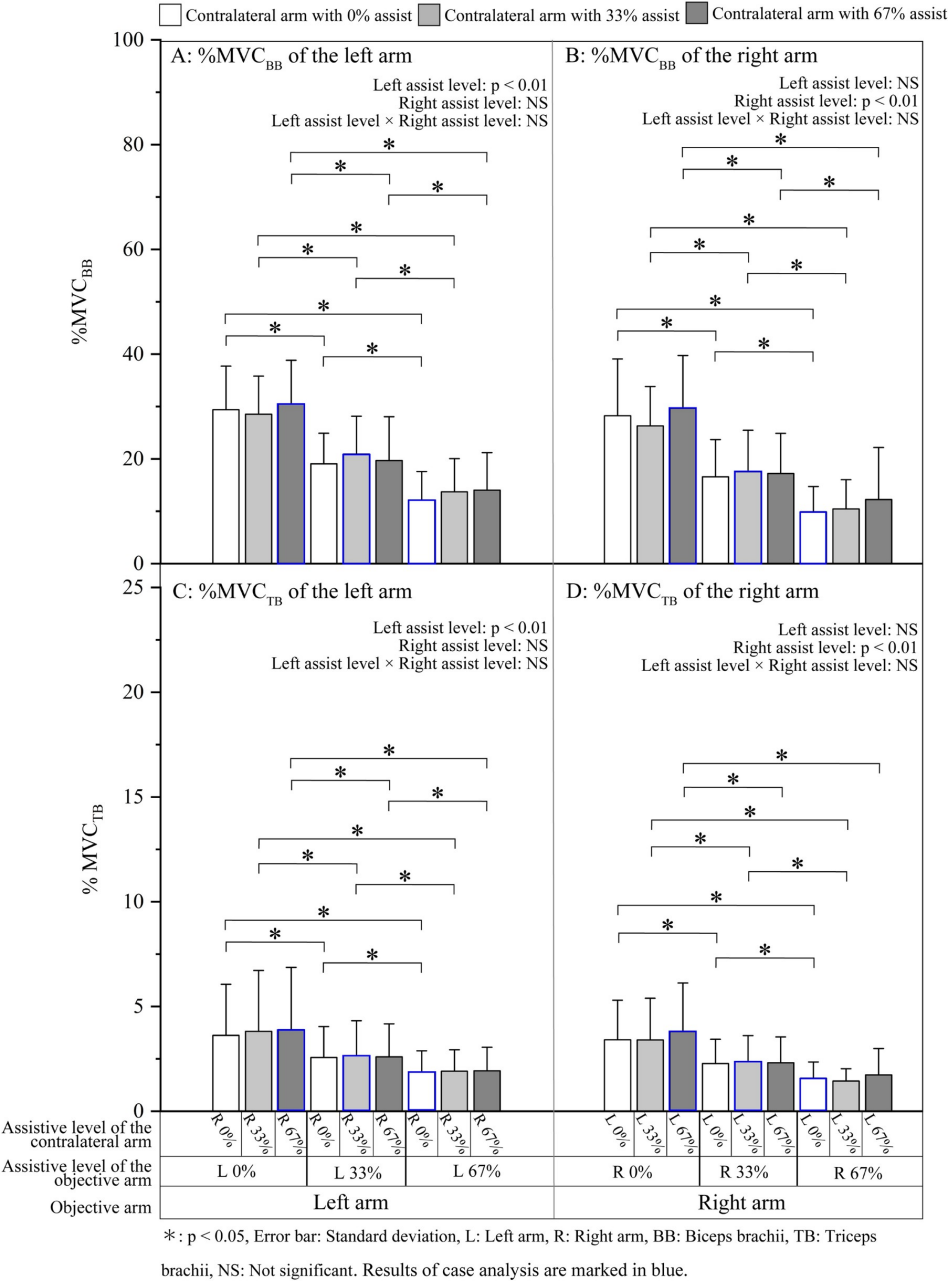
Mean muscle activity of bilateral biceps brachii and triceps brachii during the steady period.

*%MVC*_*BB*_ *and %MVC*_*TB*_. The mean %MVC_BB_ and %MVC_TB_ values during steady periods for the left and right arms are shown in Fig 4. Statistical results showed a significant main effect of the assistive force applied to the objective arm, whereas the main effect of the assistive force applied to the contralateral arm and the left assistive level × right assistive level interaction were not significant (Table 3). The results of the post hoc tests are shown in Fig 4, with asterisks indicating a significant difference between the bilateral assistive conditions. In general, the %MVC_BB_ and %MVC_TB_ of both arms showed a tendency to decrease with the intensity of the assistive force applied to the objective arm, whereas maintained a certain level regardless of the increase in assistive force applied to the contralateral arm.

**Fig 5:**
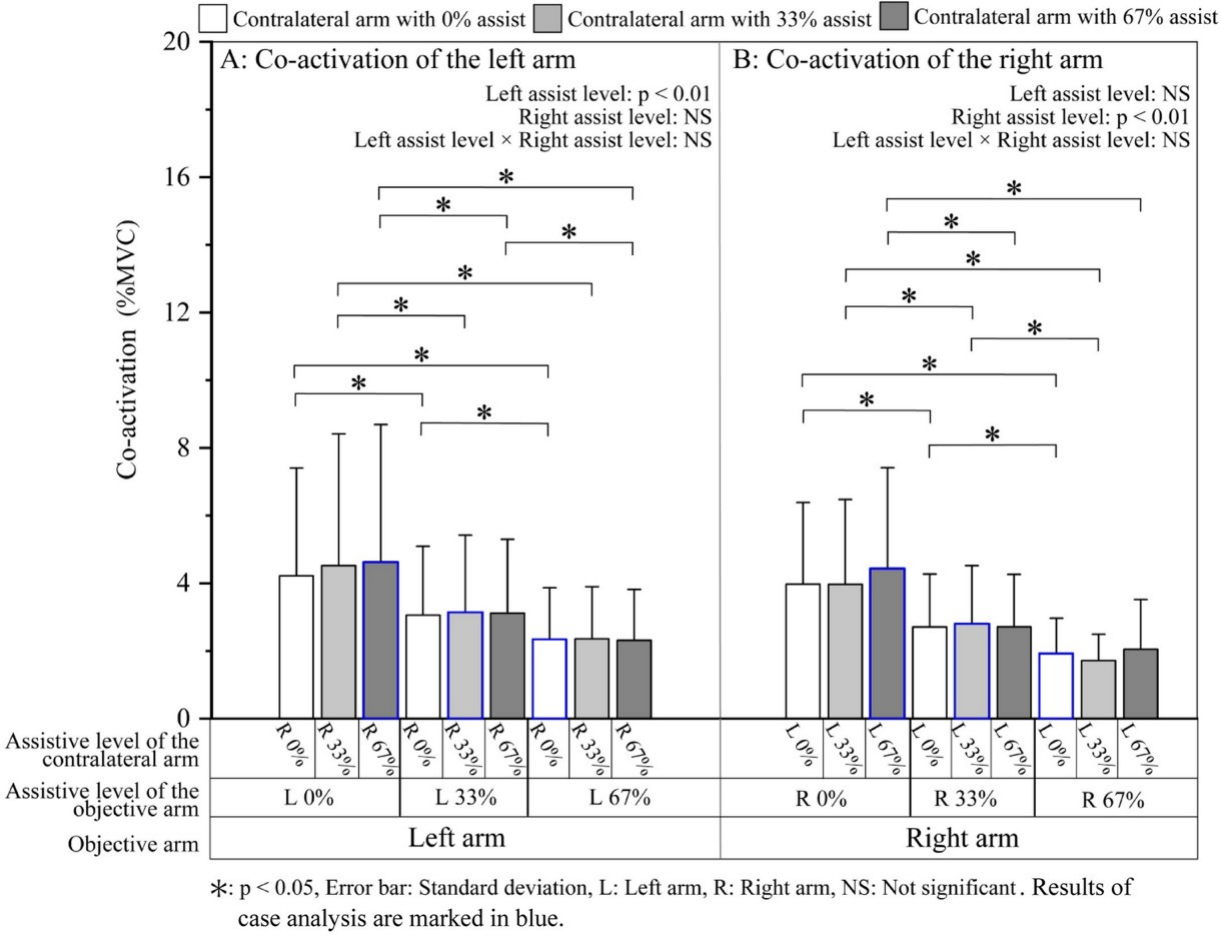
Co-activation of (A) the left and (B) right arms during the steady period.

*Co-activation*. The mean co-activations of the two arms under each assistive condition are shown in Fig 5. Similar to %MVC_BB_ and %MVC_TB_, co-activation level of the objective arm only significantly decreased with the increase in the assistive level applied on itself. No significant main effects of the assistive level of the contralateral arm and left assistive level × right assistive level interaction were found (Table 3).

**Fig 6:**
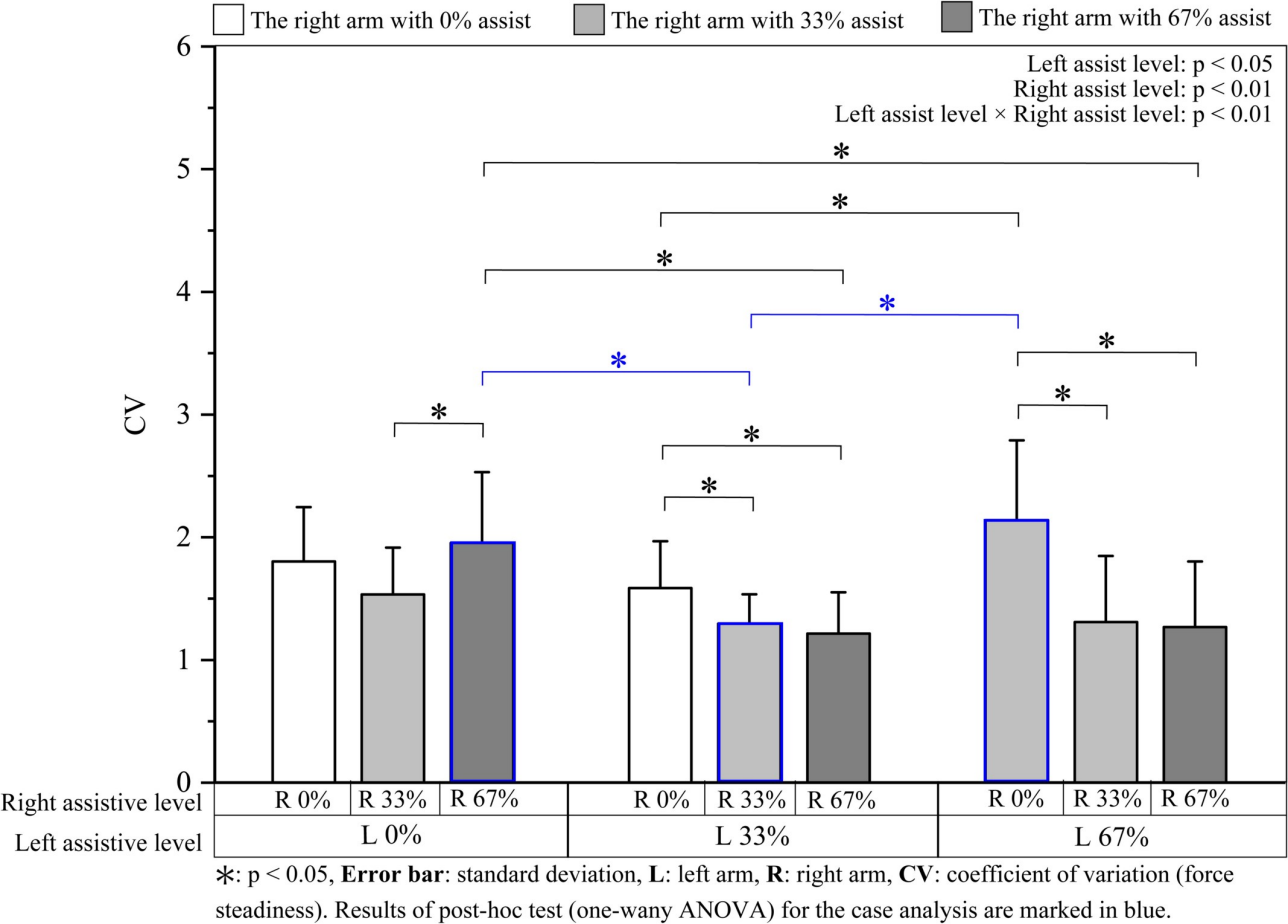
Bilateral force steadiness (CV) during the steady period.

**Fig 7:**
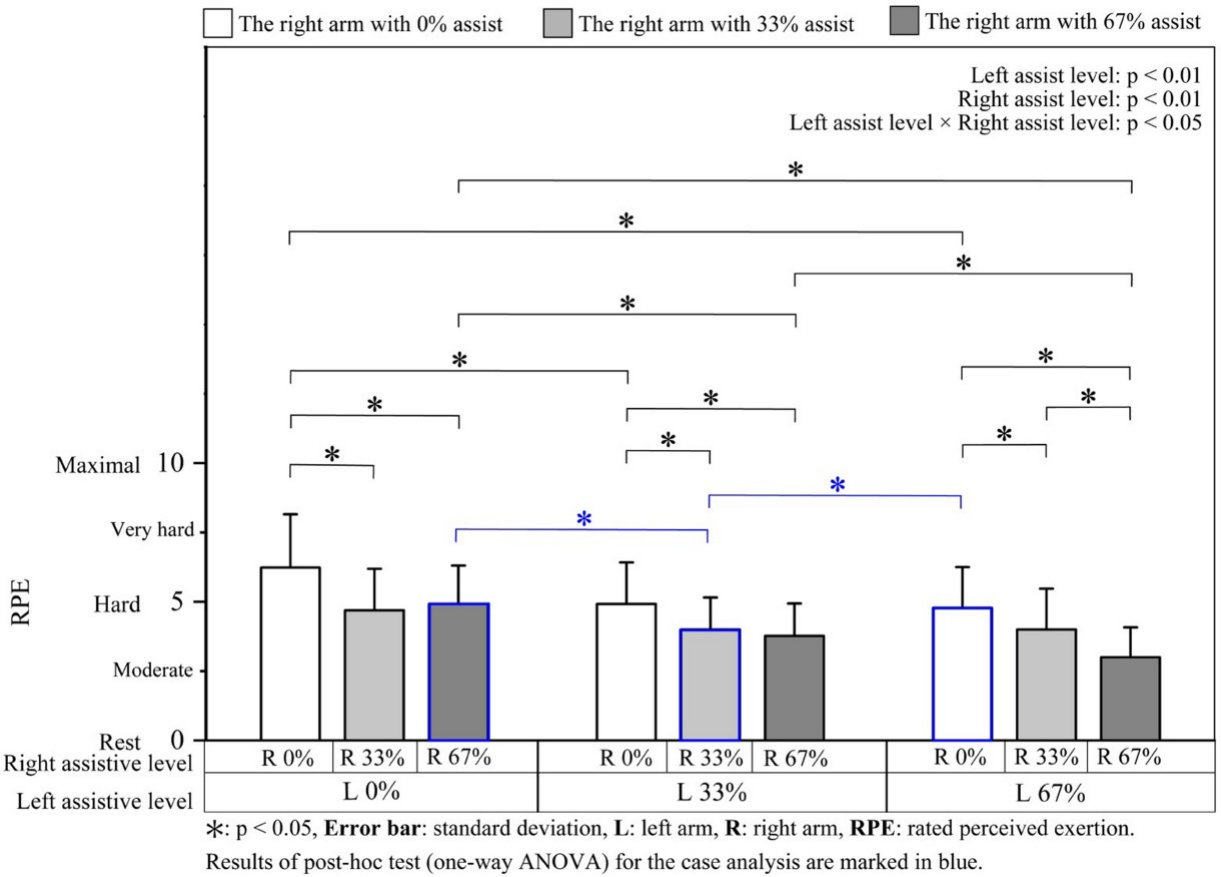
RPE under different assistive conditions.

*Force steadiness (CV) and rated perceived exertion (RPE)*. Mean CV and RPE during the steady state are presented in Figs 6 and 7, respectively. For the bilateral differences in CV and RPE, the results of the one-way ANOVA (9 bilateral assistive conditions) showed a significant main effect of the bilateral assistive condition (CV: F [8, 96] = 9.45, p < 0.01; RPE: F [3.74, 44.88] = 22.43, p < 0.01, ε = 0.47). Among the results of the post hoc tests, we focused on 3 pairs of assistive conditions that have bilateral interchangeable assistive levels; we found no significant difference between L0% & R33% and L33% & R0%, L0% & R67% and L67% & R0%, and L67% & R33% and L33% & R67% (for all three pairs, CV: p > 0.99, RPE: p > 0.99).

For the bilateral interference (two-way ANOVA: [3 left assistive levels × 3 right assistive levels]), both of the CV and RPE were significantly affected by the assistive levels of both the left and the right arms (Table 3). The results of the post hoc tests are shown in Figs 6 and 7 with asterisks. RPE decreased with the increase in left assistive levels and decreased even more with the increase in the intensity of the right assistive levels. In general, RPE was alleviated with an increase in bilateral assistive force, with maximum and minimum values at L0% & R0% and L67% & R67%, respectively (Fig 7).

## Discussion of the overall results

The overall results showed no significant effects of handedness on %MVC_BB_, %MVC_TB_, co-activation, CV, and RPE; therefore, the two arms may be equally adaptable to the bilateral assistance. We anticipated a lower muscle activity of the dominant arm due to its advantage in motor control (i.e., higher mechanical effectiveness of motor units [18]). However, the results of 9 pairs of bilateral comparison (Table 2) showed that the objective arms that were assisted with the same level at bilateral interchangeable assistance pairs, regardless of whether it was the dominant or the non-dominant arm, adjusted the muscle activities and co-activation to similar levels (Figs 4 and 5). The similar bilateral muscular responses could be explained through two aspects: from the perspective of the movement task, previous studies that revealed bilateral differences in motor control strategy mainly tested unilateral reaching movements [19, 28, 29] and the force-matching task [18] during which each arm performed an independent motion. In the bilateral force-matching task in this study, which mimicked a weight-holding scenario, the two arms were connected through a rod so that the motion of the two arms could directly interact with each other. This bilateral interaction might undermine the advantage of the dominant arm in motor control, and together with the effect of the angular restriction of the rod, both arms might ultimately have coordinated in a similar pattern at bilateral interchangeable assistance pairs and thus did not show the bilateral differences in motor control that were observed in previous studies. On the other hand, even though there was rod restriction during the task, the two forearms were free to move, so the two-target task needed bilateral cooperation to match the target force and the inter-coordination of the two arms (i.e., coordination in timing and activation of bilateral homologous muscles) to quell the disturbance caused by external forces. From the perspective of motor control, a similar level of muscle activities suggests that the two arms might have adopted a similar motor and muscular adjustment mechanism to accommodate to the bilateral assistive forces and to achieve interlimb coordination during the two-target task.

Against hypothesis 2, the results showed that muscle activity of the objective arm was only affected by the applied assistive force to the objective arm but not the contralateral arm. Bilateral interference during the force-matching task (without direct interaction between arms) might result from two kinds of control mechanisms: (1) the interactions of the independent controls of the two arms [30] when the dominant (right) arm performed the task with a higher force production [17], such as L33% & R0% and L67% & R33% in this study, and (2) the control of the two arms as a single unit [14] when the non-dominant (left) arm performed the task with a higher force production [17] (i.e., L0% & R33% and L33% & R67%). Both control mechanisms would be adopted during the experimental tasks. However, to meet the angular restriction of the rod, the two arms might be mainly controlled independently to adjust the height of each end of the rod. In addition, the rod evenly distributed the workload to the two arms during the steady period, and its angular restriction limited the posture of both arms. As a result, the arm that was assisted at a constant level (i.e., the left arm at assistive conditions of L33% & R0%, L33% & R33%, and L33% & R67%) held the same weight with the same level of assistance in the same arm position, which might have resulted in a similar level of muscle activity. Therefore, similar to a previous study [19] that suggested that the movements of the two arms occur through different control systems, this result indicates that each arm might be capable of manipulating motor control processes independently without significant interference from the contralateral arm during the weight-holding scenario.

With regard to force steadiness and RPE, a similar force steadiness and RPE score were observed between the pairs of bilateral interchangeable assistance (Figs 6 and 7). For example, L0% & R33% and L33% & R0% (as well as L0% & R67% and L67% & R0%), two unilateral assistances, showed similar CV and RPE score, regardless of whether perturbance was applied to the dominant or non-dominant arm; the two asymmetrical assistances: L33% & R67% and L67% & R33% also showed similar values of CV and RPE regardless of which arm the higher assistive force was applied to. In light of the similar muscular responses at these bilateral interchangeable assistances, the similar muscular adjustment strategy adopted by both arms might have enabled them to overcome the differences (i.e., different external forces) between the two arms and to perform the force-matching tasks with similar accuracies. Furthermore, in an earlier study [31], we found that the non-dominant arm could maintain a force with a higher accuracy during assisted unilateral movements. However, no such advantage for the non-dominant arm was observed in the bilateral movements. Corresponding to the result of muscle activity, the coordination of both arms might diminish the inter-limb differences and undermine the advantages and disadvantages of the arms.

Finally, a post hoc power analysis was conducted using Gpower [32] (n = 13, α = 0.05, d = 0. 50) and it revealed a statistical power close to 0.50. This may have played a role in limiting the significant effects of some of the condition comparisons conducted. A sample size of approximately 27 participants would be needed to obtain statistical power at the recommended 0.80 level [33].

As unilateral assistance is a form of asymmetrical assistance, we compared task performances, including muscle activity, CV, and RPE of the unilateral and symmetrical assistances to test hypothesis 3.

### Case analysis: A comparison of assistive conditions with similar overall workloads (L0% & R67%, L33% & R33%, and L67% & R0%)

Three out of nine assistive conditions in this study had similar values of overall assistive force and represent the various ways of distributing a 67% overall assistive force to two arms (L0% & R67%, L33% & R33%, and L67% & R0%). These assistive conditions can be categorized as 67% unilateral assistance (L0% & R67% and L67% & R0%) and 33% symmetrical bilateral assistance (L33% & R33%). The two arms might have various physiological responses to different patterns of assistance. Accordingly, the aim of this case analysis was to investigate the performance differences between the 33% symmetrical assistive condition and the 67% unilateral assistive condition. Since the emphasis was on the overall performance of both arms, we analyzed the overall (summed) muscle activity of the left and the right arms.

#### Case analysis results

**Fig 8:**
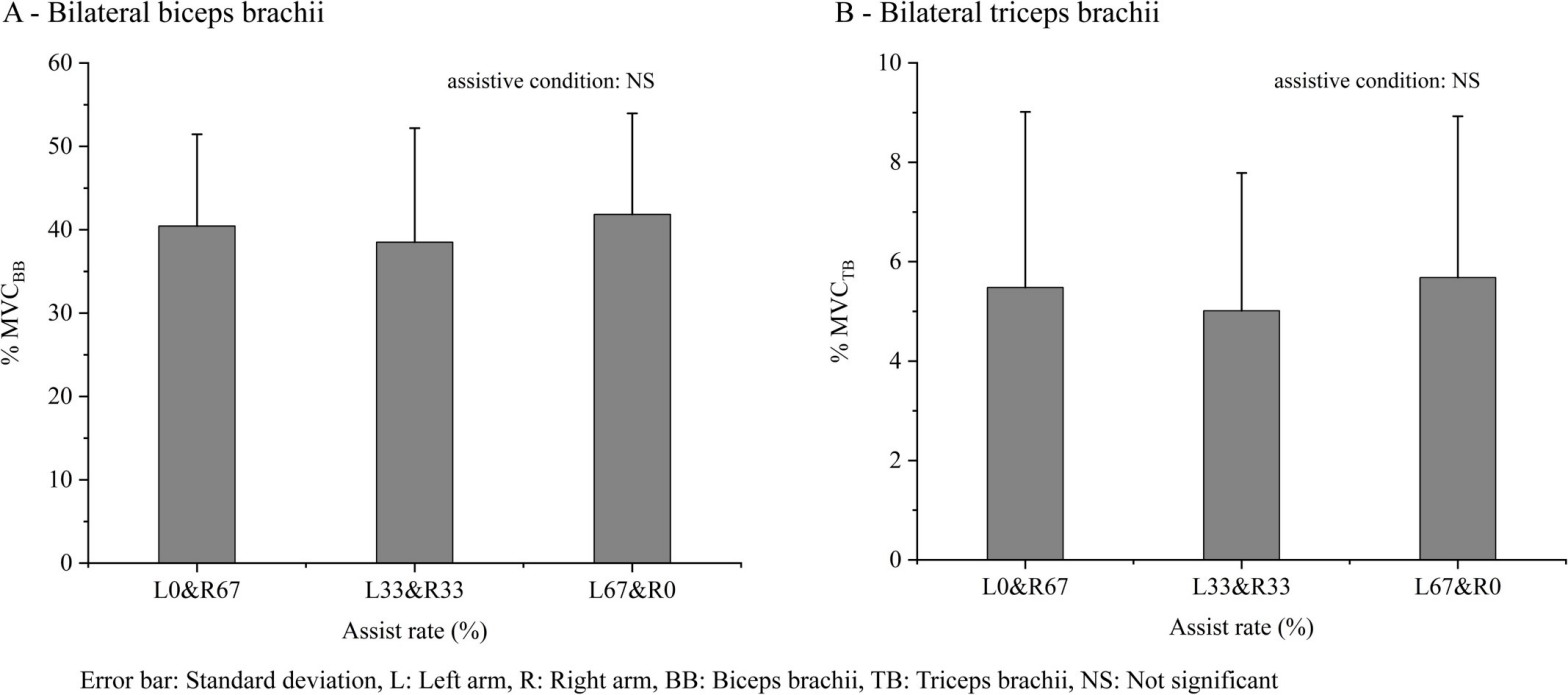
The overall muscle activity of (A) BB and (B) TB at various assistive conditions with the same overall workload.

*Muscle activity of bilateral BB and TB*. The overall muscle activity at the three assistive conditions are shown in Fig 8: (A) For BB and (B) for TB. The results of the one-way ANOVA (3 assistive conditions) showed no significant main effect of the assistive condition on the overall %MVC_BB_ (F [2, 24] = 1.28, p = 0.296) or overall %MVC_TB_ (F [2, 24] = 2.65, p = 0.091).

*Force steadiness*. The post hoc tests for one-way ANOVA (9 bilateral assistive conditions) showed that CV under the 33% symmetrical assistive condition was significantly smaller than that under the 67% unilateral assistive conditions (compared with L0% & R67%: p < 0.05; compared with L67% & R0%: p < 0.05; Fig 6, marked in blue). No significant difference was found between L0% & R67% and L67% & R0% (p > 0.99).

*Rated perceived exertion*. Similar to the CV results, the results of the post hoc tests for one-way ANOVA (9 bilateral assistive conditions) showed significant differences in RPE when comparing L33% & R33% to L0% & R67% (p < 0.05) and L67% & R0% (p < 0.05), whereas no significant differences were found between L0% & R67% and L67% & R0% (p > 0.99) (Fig 7, marked in blue).

#### Case analysis discussion

*Bilateral arm muscle activity*. The overall %MVC_BB_ and %MVC_TB_ under the assistive conditions of L0% & R67%, L33% & R33%, and L67% & R0% showed similar values (Fig 8), indicating that both 33% symmetrical assistance and 67% unilateral assistance have a similar effect of alleviating the workload.

*Force steadiness*. Studies on the unilateral tasks indicated that the CVs increase as the level of force production increase [34, 35]. Therefore, the CVs could theoretically maintain levels consistent with the force production. In our study, although the overall force production of both arms under three bilateral assistive conditions were the same, a larger CV value was observed under the 67% unilateral assistive conditions compared with that under the 33% symmetrical bilateral assistive condition (Fig 6). Noted that the difference in external assistive forces between arms was larger at the 67% unilateral assistive condition. Accordingly, force steadiness during bilateral force-matching tasks with assistance might not only be affected by the overall force production of both arms but also by the relative difference of assistive forces between the arms. Two possible reasons for this result include:

#### (1) *Different bilateral coordination patterns during the two assistive patterns*

At L33 & R33 assistance, bilateral homologous muscle groups were recruited at a similar level simultaneously throughout the force-matching task due to the two arms being assisted at the same level. Therefore, both arms might have coordinated in an in-phase pattern at symmetrical assistance. At 67% unilateral assistance, the unassisted arm activated a higher level of muscle activity of the BB and TB muscles than the assisted arm. The high neural signal magnitude caused a large noise in motor comments [36], resulting in a higher force variability in the unassisted arm. The asymmetry in force variability between arms might have affected bilateral force steadiness. Additionally, the assisted arm and unassisted arm might be adjusted independently to overcome the external-force-caused perturbation. In order to adjust the rod back to horizontal, the movement of one arm (possibly the unassisted arm) might be suppressed to compensate for the asymmetry caused by the unilateral assistive force, which is different from the in-phase mode adjustment. Corresponding to a finding of previous studies, in-phase movements may be performed more efficiently and effortlessly compared with anti-phase movements [37, 38], or movements with different activation levels of bilateral homologous muscle groups, in the case of unilateral assistance.

#### (2) *Different levels of bilateral interference*

It has been suggested that interactions between the two hands during bilateral movements result from multiple levels of neural cross-talk (motor overflow) between the signals controlling the two limbs [39]. As reported in a previous study [40], the motor overflow asymmetry increases with the difference in force amplitude between arms. Consequently, a stronger bilateral interference is observed when the degree of force amplitude asymmetry increases between arms. In the present case, the 33% symmetrical bilateral assistive condition might have maintained a timing-consistent activation of homologous muscles. It is possible that the signals of both contralateral and ipsilateral descending pathways were congruent [39], thereby resulting in a more stable bilateral control. On the other hand, under both L0% & R67% and L67% & R0% assistive conditions, the greater difference in assistive force between both arms might have increased the cross-talk between the hemispheres of the brain, thus inducing a stronger bilateral interference, which might have deteriorated the force steadiness during bilateral force-matching tasks.

*Rated perceived exertion*: RPE is related to exercise intensity and the main working muscle activity [41]. However, although the overall %MVC_BB_ were similar under assistive conditions of L0% & R67%, L33% & R33%, and L67% & R0%, the RPE was greater under the 67% unilateral assistive condition than under the 33% symmetrical bilateral assistive condition (Fig 7), which was similar to the CV results. The maximum rod angle deviations immediately after the application of assistive force were 3.65°, 0.91°, and 2.32° at the three assistances, respectively (Fig B in S1 Fig). The relatively smaller rod angle deviation at L33% & R33% suggests that the participants might not have spent much effort adjusting the rod angle, thus enabling them to focus only on the force matching. Therefore, the task with symmetrical assistance might have been easier to perform than the one with unilateral assistance. Besides, the two arms may be more adaptable at controlling bilateral force exertions under symmetric assistive conditions (i.e., the in-phase movement may be performed with less attentional load) compared with unilateral assistive conditions.

In general, symmetrical bilateral assistive conditions resulted in a steadier force control with less effort than unilateral assistive conditions, which is in line with hypothesis 3.

### Limitations and future studies

We only measured the total force production of the two arms during the force-matching tasks. The measurement of force produced by each arm would be helpful in analyzing the synchronization of the two arms. Our study was also limited by experimental participants (only healthy male), movement form (static and isometric contraction), workload intensity (30%MF), and duration (30 s). If these factors were to be changed, the physiological responses for both arms might be different. Further research is necessary to examine the specific responses of arms during these scenarios.

## Conclusion

The overall result of the 9 assistive conditions showed a similar muscular response of the two arms to bilateral assistive conditions. Considering that the muscle activity of the objective arms only decreased with the increase in the assistive force that was applied on itself, we concluded that both arms might have adopted a similar but independent motor control mechanism to acclimate to the bilateral external assistive forces. Among the nine assistive conditions, three pairs of bilateral assistive conditions that have bilateral interchangeable assistive levels, including two pairs of unilateral assistive conditions—L0% & R33%, and L33% & R0%, L0% & R67%, and L67% & R0%, and one pair of symmetrical assistive condition—L33% & R67%, and L67% & R33%. The similar muscular response, CV, and RPE in these pairs indicated that bilateral assistances with interchanged assistive levels generally had similar effects on muscle activity reduction, regardless of the arm to which the higher or lower assistive force was applied.

The comparison of L0% & R67%, L33% & R33%, and L67% & R0% indicated that: (1) both 33% symmetrical assistance and 67% unilateral assistance had similar effectiveness in reducing muscle activities; and (2) the participants could control bilateral force production more accurately under the 33% symmetrical assistive condition than under the 67% unilateral assistive condition, suggesting that better force steadiness would be obtained more effortlessly when the assistive force is distributed evenly to both arms rather than when the assistive force is applied to only one arm.

The findings of this research might provide insight into the control and assisting method of PASs when performing bilateral upper limb movements. The requirements for a PAS vary depending on the specific scenarios; for example, it might be better for a two-handed PAS to provide both arms with the same intensity of assistance (symmetrical) when performing bilateral tasks that require high movement stability, and it might not be necessary to consider the bilateral differences when using a one-handed PAS because equipping either arm with the PAS would result in similar task performance.

## Supporting information

S1 Raw data (PDF)

S1 Fig (ZIP)
