# Supplementary material for: Motor control characteristics of upper limbs in response to assistive forces during bilateral tasks
Source: PLoS One. 2021 Jan 7;16(1):e0245049. doi: 10.1371/journal.pone.0245049 (PMC7790287; doi:10.1371/journal.pone.0245049)
Supplement: S1 Raw data — (PDF) [file pone.0245049.s001.PDF]

Participants information

Participant ID	Age (year)	Height (cm)	Weight (kg)	Left arm (cm)		Right arm (cm)	
				Upper arm length	Forearm length	Upper arm length	Forearm length
ID1	29	180.1	72.8	31.9	25.2	32.5	25.5
ID2	23	171.5	58.9	34.2	24.6	33.8	25.1
ID3	23	166.1	64.5	30.6	21.9	30.1	21.4
ID4	23	179.9	53.4	33.1	23.1	32.4	23.6
ID5	24	181.1	69.7	32.8	24.3	34.3	25.2
ID6	27	182.3	77.2	32.8	23.8	32.7	24.1
ID7	23	170.7	64.0	32.1	22.1	31.3	22.6
ID8	23	178.9	66.0	32.8	22.1	32.3	22.0
ID9	23	166.7	61.7	31.3	22.9	31.1	22.9
ID10	24	179.5	73.2	34.5	23.9	33.4	23.9
ID11	24	170.2	57.0	31.4	21.1	31.0	21.5
ID12	24	164.1	56.5	29.9	21.0	29.9	21.4
ID13	24	179	81.8	32.9	23.1	32.6	23.8

MVC task

Participant ID	Left			Right		
	Biceps (mV)	Triceps (mV)	MVF (kgf)	Biceps (mV)	Triceps (mV)	MVF (kgf)
ID 1	0.539	0.201	16.5	0.386	0.333	18.6
ID 2	0.334	0.210	14.1	0.324	0.223	17.1
ID 3	0.390	0.145	16.5	0.321	0.185	14.7
ID 4	0.493	0.497	14.5	0.302	0.494	15.4
ID 5	0.683	0.282	19.6	0.842	0.336	21.0
ID 6	0.560	0.116	24.0	0.657	0.254	24.8
ID 7	0.153	0.239	13.7	0.162	0.198	13.3
ID 8	0.260	0.236	13.8	0.282	0.250	15.2
ID 9	0.485	0.275	17.8	0.628	0.229	19.8
ID 10	0.206	0.231	17.0	0.132	0.168	15.1
ID 11	0.191	0.091	10.9	0.205	0.128	12.8
ID 12	0.334	0.269	17.8	0.321	0.300	18.1
ID 13	0.200	0.136	17.1	0.270	0.162	20.7

MF task

Participant ID	Maximum force (kgf)	Rod angle (°)	(%MVC)			
			Left		Right	
			Biceps	Triceps	Biceps	Triceps
ID 1	35.3	-2.2	95.2	8.1	90.8	5
ID 2	31.6	4.1	100.2	10.2	81	6.1
ID 3	31.1	-5	84.4	16.2	82.4	17.7
ID 4	33.4	9.4	80.9	3.8	114.6	4.4
ID 5	38.5	-1	115	9.2	96.1	6.6
ID 6	47.7	-1.4	146	23.5	128	15.3
ID 7	25.8	-0.9	112.1	9.1	132.8	14.7
ID 8	23.5	-1.8	83.6	5.5	75.1	4.7
ID 9	35.6	-2.1	79	7.5	92.2	10.6
ID 10	30.3	1	129.5	6.8	73.5	12.2
ID 11	22.5	-1.8	101.2	25.3	94.6	14.8
ID 12	33.5	-1.4	84.6	5.1	111.2	7.2
ID 13	35.9	0.1	111.1	14.1	59.8	10.7

The rectified EMG - seatdy state of force matching task

Mean of the last 10 seconds (20-30s)	left-biceps-rectified (%MVC)								
	Bilateral assist conditions L%-R%								
	0-0	0-33	0-67	33-0	33-33	33-67	67-0	67-33	67-67
ID 1	15.0	16.4	26.1	10.7	9.7	9.9	3.4	4.9	4.4
ID 2	31.8	33.8	30.6	21.3	23.0	20.7	15.5	13.5	14.5
ID 3	26.0	20.7	19.8	11.2	13.1	13.7	4.1	5.0	5.9
ID 4	22.8	24.4	32.5	12.4	14.0	12.9	9.4	9.0	7.4
ID 5	21.1	18.1	20.9	15.2	15.1	14.5	10.1	8.3	7.6
ID 6	26.8	31.7	37.2	16.6	18.1	11.4	10.2	9.4	10.7
ID 7	32.4	27.5	27.7	26.9	26.9	28.1	15.8	18.2	17.0
ID 8	29.3	29.9	39.4	23.8	31.5	34.3	17.7	23.6	19.9
ID 9	21.0	24.9	21.4	14.7	15.2	15.5	6.1	9.2	8.8
ID 10	43.7	37.6	39.4	23.6	31.8	29.4	19.5	25.3	22.6
ID 11	38.6	38.1	33.7	20.9	22.9	22.2	12.7	13.5	17.9
ID 12	36.9	36.6	33.8	24.2	27.0	24.7	15.1	12.9	15.4
ID 13	37.0	31.5	42.3	25.8	23.6	23.8	18.6	20.7	20.4
Mean of the last 10 seconds (20-30s)	left-triceps-rectified (%MVC)								
	Bilateral assist conditions L%-R%								
	0-0	0-33	0-67	33-0	33-33	33-67	67-0	67-33	67-67
ID 1	2.3	1.9	2.1	1.7	1.5	1.7	1.2	1.3	1.3
ID 2	3.4	3.6	3.7	2.3	2.3	2.5	1.7	1.6	1.7
ID 3	5.0	5.3	5.1	4.1	3.5	4.0	2.7	2.8	2.5
ID 4	1.3	1.5	1.7	1.0	1.0	1.0	0.8	0.8	0.7
ID 5	2.4	2.0	2.0	1.6	1.7	1.6	1.2	1.1	1.2
ID 6	6.9	7.6	8.4	4.7	5.2	4.1	3.8	3.8	3.1
ID 7	2.8	2.5	2.5	2.6	2.2	2.4	1.8	1.8	1.6
ID 8	1.9	1.9	2.0	1.7	1.9	1.9	1.3	1.3	1.5
ID 9	2.5	2.8	2.5	1.5	1.8	1.7	0.9	1.0	0.9
ID 10	2.0	2.0	2.1	1.6	1.7	1.7	1.6	1.5	1.5
ID 11	9.9	11.6	11.6	5.9	6.8	6.8	3.9	3.8	4.8
ID 12	2.3	2.6	2.3	1.8	1.6	1.8	1.2	1.1	1.1
ID 13	4.5	4.2	4.8	3.0	3.1	3.0	2.2	2.6	2.3

Mean of the last 10 seconds (20-30s)	right-biceps-rectified (%MVC)								
	Bilateral assist conditions L%-R%								
	0-0	0-33	0-67	33-0	33-33	33-67	67-0	67-33	67-67
ID 1	17.4	9.1	6.2	15.9	9.3	5.7	23.0	7.6	5.7
ID 2	25.8	12.1	7.7	21.0	13.8	9.2	24.5	14.8	8.5
ID 3	18.8	12.6	5.7	28.2	10.6	6.3	26.6	15.1	7.1
ID 4	40.7	20.2	12.6	28.7	22.4	14.0	34.8	18.9	12.0
ID 5	17.8	8.7	3.9	18.4	7.6	4.5	18.4	10.2	3.4
ID 6	30.1	13.7	10.1	31.7	13.0	7.6	31.7	15.0	8.6
ID 7	43.5	25.6	21.1	42.0	34.5	25.0	50.7	33.7	31.1
ID 8	24.9	25.7	12.8	27.2	23.9	12.9	27.8	21.7	13.6
ID 9	20.4	9.8	6.8	27.0	14.1	4.9	25.4	10.6	5.8
ID 10	31.3	20.3	14.2	29.6	21.9	15.3	26.5	20.4	18.7
ID 11	32.5	22.8	10.8	36.4	24.3	11.4	36.7	25.0	13.0
ID 12	48.8	26.8	12.3	38.6	22.5	11.0	45.0	23.0	13.3
ID 13	15.0	8.7	4.9	13.5	10.8	6.2	14.9	9.5	7.4

Mean of the last 10 seconds (20-30s)	right-triceps-rectified (%MVC)								
	Bilateral assist conditions L%-R%								
	0-0	0-33	0-67	33-0	33-33	33-67	67-0	67-33	67-67
ID 1	1.4	0.8	0.7	1.0	0.8	0.7	1.3	0.8	0.8
ID 2	2.3	1.6	1.1	2.1	1.5	1.2	2.4	1.7	1.2
ID 3	5.0	3.4	2.1	6.4	3.7	2.2	6.5	4.0	2.4
ID 4	1.4	0.9	0.7	1.4	1.0	0.6	1.5	1.0	0.7
ID 5	2.3	1.5	0.9	2.1	1.4	1.1	2.0	1.4	0.9
ID 6	7.5	4.6	3.3	7.0	4.8	2.0	8.4	3.8	2.5
ID 7	5.0	3.0	2.6	5.3	3.8	2.2	5.7	3.8	2.9
ID 8	1.8	1.6	1.0	2.1	1.6	1.0	1.9	1.6	1.0
ID 9	2.9	1.9	1.5	3.9	2.3	1.1	3.5	1.9	1.1
ID 10	2.6	1.9	1.5	2.4	2.0	1.6	2.7	1.8	1.6
ID 11	5.9	4.0	2.2	6.3	3.9	2.3	7.0	4.3	2.4
ID 12	2.7	1.9	1.0	2.8	1.6	1.0	3.1	1.7	1.1
ID 13	3.5	2.5	1.8	3.9	2.5	1.7	3.4	2.4	1.6

Force steadiness - steady state of force matiching task

Bilateral assist conditions		0-0	0-33	0-67	33-0	33-33	33-67	67-0	67-33	67-67
Force steadiness (CV)	ID1	1.83	1.2	1.78	1.45	0.91	1.29	3.33	2.31	0.53
	ID2	1.74	1.62	2.36	1.55	1.3	1.1	1.67	1.26	1.56
	ID3	2.12	1.43	2.12	1.9	1.39	0.88	2.24	0.73	1.52
	ID4	1.69	1.68	1.82	1.79	1.35	1.35	2.36	1.45	1.26
	ID5	0.91	0.95	1.31	0.83	1.09	0.93	1.05	0.65	0.54
	ID6	1.46	1.1	1.19	1.25	1.27	0.64	1.93	0.83	0.91
	ID7	2.75	1.88	2.04	1.61	0.94	1.39	1.86	1.78	0.9
	ID8	1.86	1.46	1.85	1.97	1.78	1.58	2	1.18	2.25
	ID9	1.71	1.46	1.59	2.26	1.5	1.79	2.34	1.2	2.17
	ID10	1.89	1.42	1.62	1.11	1.14	1.09	2.08	0.8	1.02
	ID11	2.16	1.56	3.29	1.76	1.54	1.67	3.33	2.28	1.2
	ID12	1.32	1.71	1.69	1.72	1.29	1.14	2.25	1.2	1.08
	ID13	2	2.48	2.75	1.41	1.34	0.95	1.37	1.34	1.54

Rated perceived exertion

Bilateral assist conditions		0-0	0-33	0-67	33-0	33-33	33-67	67-0	67-33	67-67
Rated perceived exertion (1-10)	ID1	6	3	4	3	3	3	4	4	2
	ID2	9	7	7	7	6	6	8	7	5
	ID3	6	7	6	6	4	4	5	4	3
	ID4	4	4	4	4	2	2	4	2	2
	ID5	7	5	5	5	5	5	5	4	3
	ID6	4	4	4	4	4	2	3	3	3
	ID7	6	5	4	5	3	3	4	3	2
	ID8	5	4	5	5	4	4	4	4	3
	ID9	7	5	7	6	5	4	6	4	3
	ID10	5	3	3	4	3	4	3	3	2
	ID11	8	3	4	3	3	3	5	3	2
	ID12	4	4	4	4	3	4	4	4	3
	ID13	10	7	6	8	6	5	7	7	4
