# Supplementary figures and images for: Motor control characteristics of upper limbs in response to assistive forces during bilateral tasks
Source: PLoS One. 2021 Jan 7;16(1):e0245049. doi: 10.1371/journal.pone.0245049 (PMC7790287; doi:10.1371/journal.pone.0245049)

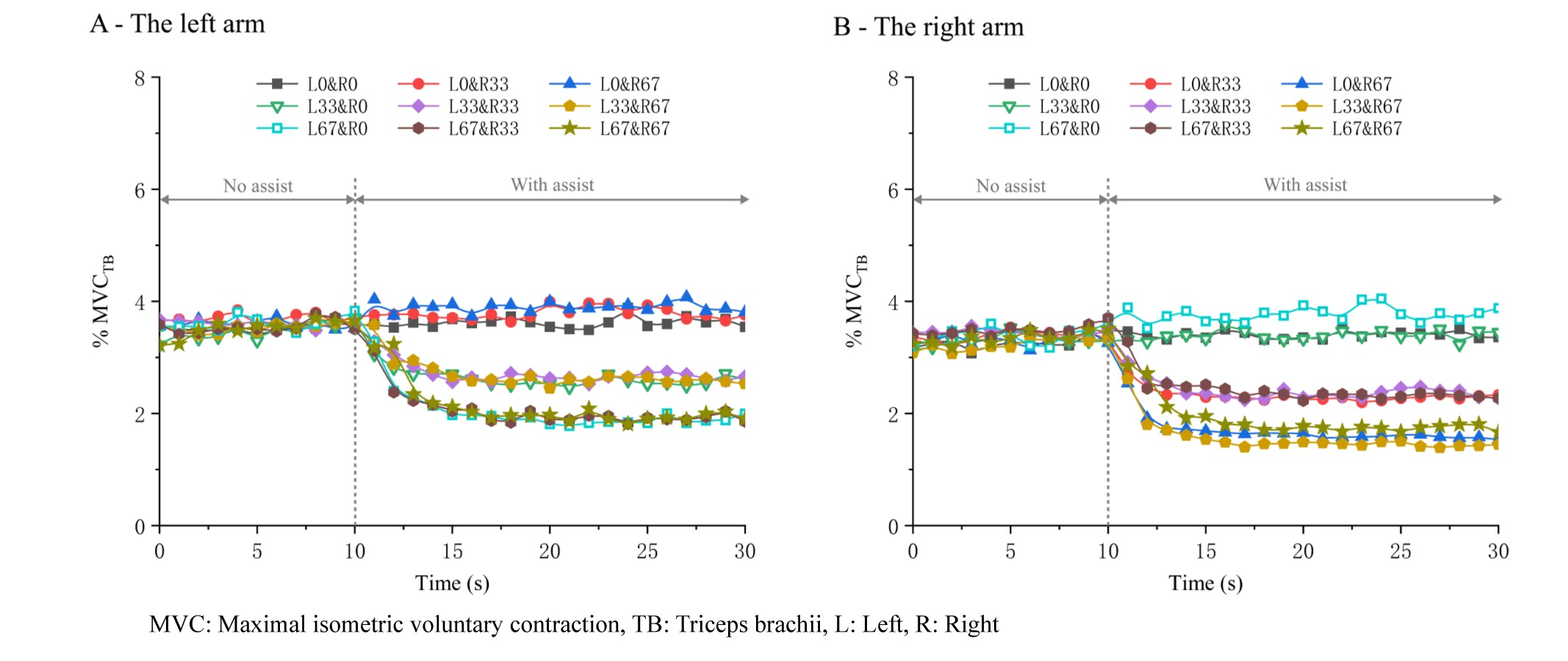

Supplement: S1 Fig — (ZIP) [file pone.0245049.s002.zip › FigA.tif]

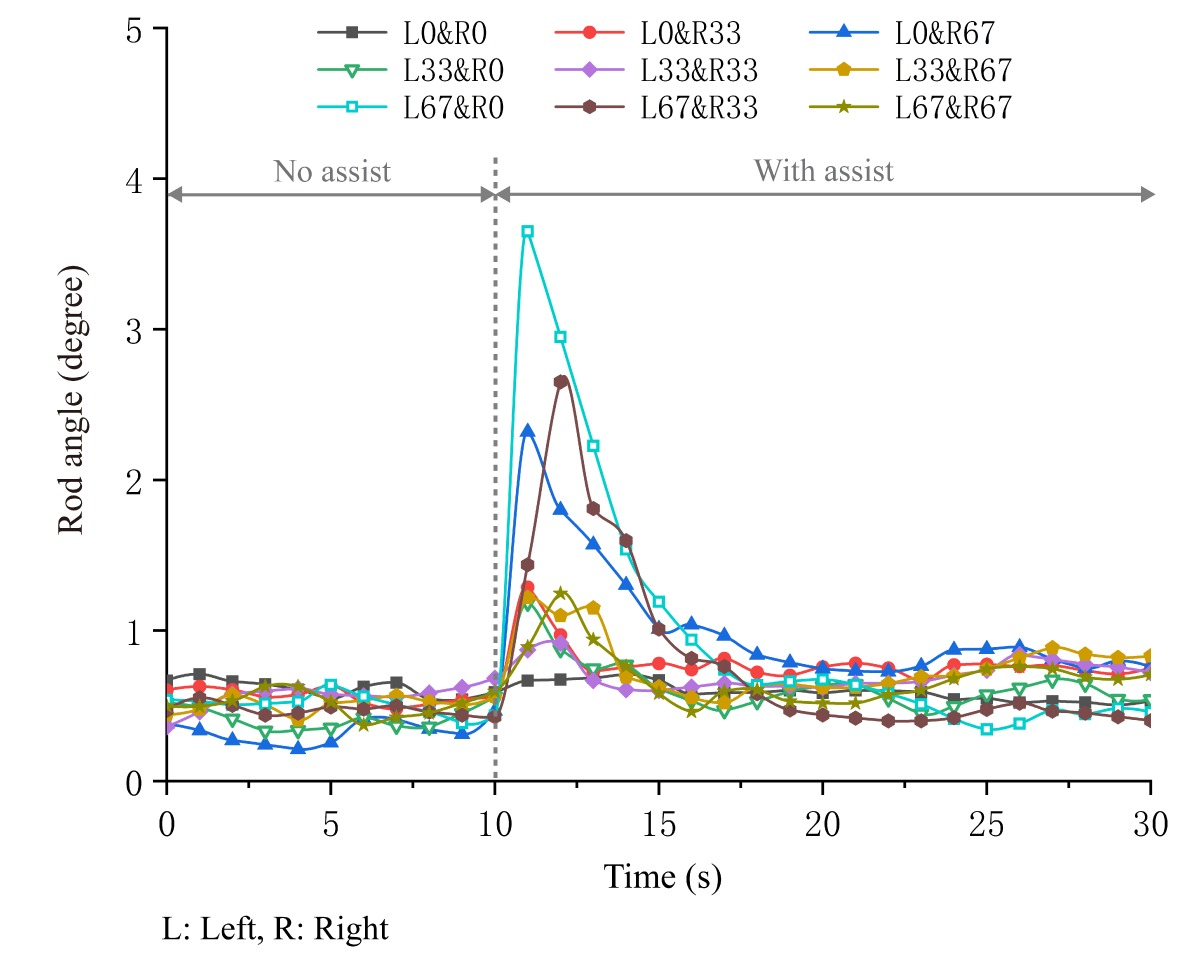

Supplement: S1 Fig — (ZIP) [file pone.0245049.s002.zip › FigB.tif]
